# Red Blood Cell Storage with Xenon: Safe or Disruption?

**DOI:** 10.3390/cells13050411

**Published:** 2024-02-27

**Authors:** Ekaterina Sherstyukova, Viktoria Sergunova, Snezhanna Kandrashina, Aleksandr Chernysh, Vladimir Inozemtsev, Galina Lomakina, Elena Kozlova

**Affiliations:** 1 Federal Research and Clinical Center of Intensive Care Medicine and Rehabilitology, V.A. Negovsky Research Institute of General Reanimatology, 107031 Moscow, Russia; 2 Faculty of Chemistry, Lomonosov Moscow State University, Lenin Hills 1/3, 119991 Moscow, Russia; 3 Department of Medical and Biological Physics, Sechenov First Moscow State Medical University (Sechenov University), 119991 Moscow, Russia

**Keywords:** xenon, blood storage, red blood cells, membrane, AFM, spectrophotometry, deoxyhemoglobin, in vitro study

## Abstract

Xenon, an inert gas commonly used in medicine, has been considered as a potential option for prolonged preservation of donor packed red blood cells (pRBCs) under hypoxic conditions. This study aimed to investigate how xenon affects erythrocyte parameters under prolonged storage. In vitro model experiments were performed using two methods to create hypoxic conditions. In the first method, xenon was introduced into bags of pRBCs which were then stored for 42 days, while in the second method, xenon was added to samples in glass tubes. The results of our experiment showed that the presence of xenon resulted in notable alterations in erythrocyte morphology, similar to those observed under standard storage conditions. For pRBC bags, hemolysis during storage with xenon exceeded the acceptable limit by a factor of six, whereas the closed-glass-tube experiment showed minimal hemolysis in samples exposed to xenon. Notably, the production of deoxyhemoglobin was specific to xenon exposure in both cell suspension and hemolysate. However, this study did not provide evidence for the purported protective properties of xenon.

## 1. Introduction

Blood cell transfusion is a critical intervention in severe blood loss, trauma, anemia, cancer treatment, and sepsis [1,2,3,4,5,6]. According to current guidelines, packed red blood cells (pRBCs) have a maximum shelf life of 42 days under certain conditions. However, this time frame is insufficient to ensure the preservation of high-quality blood units. Prolonged storage causes structural and functional changes in pRBCs known as “storage lesions” [7,8,9]. These lesions are primarily caused by the development of oxidative stress. Therefore, preservation and improvement of donor RBC quality remain important issues.

To address this issue, experts in the scientific community are actively researching alternative strategies for storing pRBCs. Antioxidants such as vitamins E, C, and beta-carotene [10] are being added to counteract the effects of storage lesions. In addition, anaerobic storage techniques are employed, including those involving the use of inert gases [11,12,13,14].

In our study, xenon (Xe) was used as an inert gas. Xe is widely used in medicine. It is an effective anesthetic and helps reduce the effects of stroke, brain injury, and lesions that cause nerve tissue necrosis [15,16]. Hyperpolarized ^129^Xe is used in MRI as a signal enhancer for human lung imaging and the study of pulmonary disease [17,18,19,20]. Xenon has been observed to have an organoprotective effect on vital organs [21,22]. In particular, xenon has been found to prolong graft survival when administered to both transplant donors (pre-treatment) and recipients (post-treatment) [23].

Like other noble gases, xenon is an odorless, colorless, single-atom gas with very low chemical activity at standard temperature and pressure [24]. Its potential as a cytoprotectant is remarkable, based on its ability to diffuse into tissues, facilitated by its compact size [25].

Hypoxic conditions using inert gases have emerged as a promising approach for long-term storage of pRBCs [12,26]. By using inert gases, the storage process is expected to minimize the occurrence of structural and biochemical changes in pRBCs caused by oxidative processes, ultimately leading to a reduction in transfusion-related complications.

Our in vitro model experiment used two methods to create hypoxic conditions. The first method involved pumping xenon into bags of pRBCs and storing them for 42 days. The second method was based on using xenon while storing the samples in glass tubes. This study aimed to investigate how xenon affects erythrocyte parameters under prolonged storage. In addition, the possible effects of erythrocyte storage in an inert gas atmosphere were studied.

## 2. Materials and Methods

### 2.1. Experimental Set-Up

**Figure 1:**
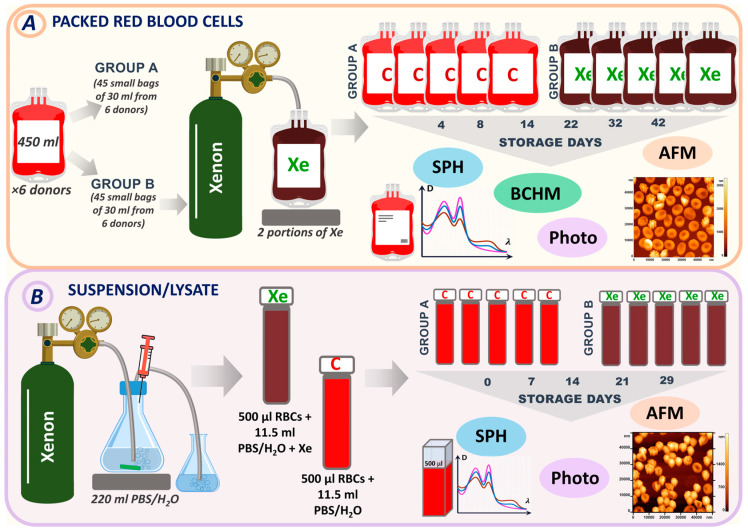
Experimental design. (**A**) Experimental design for pRBCs (method 1). (**B**) Experimental design for RBC suspension/lysate (method 2). Samples were analyzed by spectrophotometry (SPH), atomic force microscopy (AFM), biochemical assays (BCHM), and photography. Bags that did not contain xenon and were used as controls were labeled C (Group A). Bags with injected xenon were labeled Xe (Group B).

The different stages of in vitro experiments can be seen in Figure 1. In the experiment, two methods were used to create hypoxic conditions.

The first method involved pumping xenon into bags containing pRBCs. There were six donors, and each donor’s pRBCs were stored in large bags of 450 mL. These large bags were then divided into smaller bags, with 15 bags per donor. Small bags from each donor were divided into control and experimental groups (containing xenon). A total of 45 small bags containing no xenon were used as controls and labeled C (Group A). Another set of 45 small bags from different donors were stored in the presence of xenon. These bags were labeled Xe (Group B). All bags were stored under standard conditions at +4 °C for 42 days. Measurements were taken on days 4, 8, 14, 22, 32, and 42, when the corresponding bags were opened, and specific parameters were measured (Figure 1A).

The second method is based on the use of xenon during the storage of samples in glass tubes. In the second series, 220 mL of phosphate-buffered saline (PBS) (MP Biomedicals LLC, Illkirch-Graffenstaden, France) or distilled water was saturated with xenon (Figure 1B). We used PBS for the preparation of the red blood cell suspension and distilled water for the lysate. Then, 11.5 mL of the resulting solution (PBS + Xe or H_2_O + Xe) was added to 500 μL of erythrocytes in glass tubes. These samples were designated XeS for suspension and XeL for lysate. Control samples were prepared by adding PBS/H_2_O to the erythrocytes and were designated as CS for suspension and CL for lysate. All samples were stored at +4 °C for 29 days. Tubes containing suspension/lysate were opened for measurement on control days (0, 7, 14, 21, and 29).

### 2.2. Packed Red Blood Cells

Airtight bags of leukodepleted pRBCs were provided by the Moscow Blood Bank. The pRBC units were stored in JMS (JMS Singapore PTE LTD, Singapore) bags containing anticoagulant and preservation solution (CPD/SAGM). A single standard unit of pRBCs (large bag) had a volume of 450 mL. Each unit of pRBCs was dispensed in separate small bags. The volume of pRBCs in each small bag was 30 mL. Six large units of two blood groups (O (I) and A (II), of men aged 38 ± 12 years) were used in the study.

### 2.3. Donor Blood

Whole blood samples from eight healthy donors (6 males and 2 females, aged 35 ± 10 years) were collected in microbatches with EDTA during a check-up examination. Informed consent was obtained from each donor. Tubes of 10 mL of whole blood were centrifuged (2000 rpm, 5 min) to remove plasma. Subsequently, for each experiment performed, the RBCs were depleted of all white blood cells and platelets.

### 2.4. Xenon Tanks

Cylinders containing xenon gas of high purity (99.9999% pure, 100 L in volume) were acquired from AKELA-N, Russia.

To determine the dose dependence of xenon portions on the amount of deoxyhemoglobin in the suspension and lysate, PBS and H_2_O solutions were saturated with 2 to 18 xenon portions in increments of 2 portions. One xenon portion is approximately 0.8 L of compressed xenon.

The control sample with PBS was designated CS-0, and the control sample with H_2_O was designated CL-0. Xenon-saturated samples were designated as XeS-18 and XeL-18 for suspension and lysate, respectively, with the number indicating the number of xenon portions.

### 2.5. Preparing a Sample for Erythrocyte Morphology Study

A volume of 100 µL of erythrocytes was subjected to centrifugation (3000 rpm, 5 min). The supernatant was discarded. Next, 50 µL of 1% solution of glutaraldehyde (Panreac Quimica S.L.U., Barcelona, Spain) was mixed with 50 µL of cell sample. The mixture was incubated for 5 min. To avoid salt crystal formation on the microscopy slide, the specimens were rinsed with distilled water. A single layer of cells suitable for atomic force microscopy (AFM) examination was prepared using a V-Sampler (Vision, Vienna, Austria) by depositing a 10 µL droplet on a slide. The slides were examined at ambient temperature once dry.

### 2.6. Preparation of the Cytoskeleton

We have described the steps of cytoskeleton preparation in previous publications on pRBCs storage [27,28]. Briefly, 500 µL of a dilute solution (comprising 1 part 0.9% NaCl and 9 parts distilled water) was combined with 100 µL of erythrocytes. This mixture was centrifuged (3000 rpm, 5 min). The overlying liquid was discarded, leaving 75 µL of the mixture in the Eppendorf tube. Following this, 300 µL of distilled water was introduced to the 75 µL residue to promote additional red blood cell lysis. This mixture was agitated for 5 min using a Mini-Rotator Bio RS-24 (Biosan, Riga, Latvia) set at 8 rpm and then cooled in a refrigerator at +4 °C for 30 min, followed by a 10 min period at ambient temperature to further the lysis process. After another centrifugation (3000 rpm, 5 min), a layer of cell ghosts was placed onto a slide with a V-Sampler for atomic force microscopy (AFM) analysis.

### 2.7. Measuring Young’s Modulus

To prepare the suspension, 5 µL of erythrocytes was added to 10 mL of PBS. Then, 200 µL of the resulting suspension was placed onto a cover slip treated with polylysine solution (MP Biomedicals, Eschwege, Germany). The duration of adhesion to the glass surface was 40 min. The force curves were measured in a liquid environment using AFM. Consequently, all measurements of the membrane’s Young’s modulus were carried out on native cells only.

### 2.8. Atomic Force Microscopy and Spectroscopy

The NTEGRA Prima atomic force microscope (NT-MDT Spectrum Instruments, Moscow, Russia) was used to capture images of cells and their cytoskeleton. The AFM 3D images were captured with NSG01 cantilevers that had a gold reflective surface, a 10 nm tip radius, and a spring constant range of 1.45–15.1 N/m (NT-MDT Spectrum Instruments, Russia). Scanning areas varied from 100 × 100 to 1 × 1 μm^2^ in semi-contact operation mode. The resolution of each image spanned between 512 and 1024 points [29,30]. Image processing was performed using FemtoScan Online software, Version 2.3.239 (5.2) (Advanced Technologies Center, Moscow, Russia, www.nanoscopy.ru (accessed on 12 January 2023)) [31,32,33,34].

To assess the Young’s modulus of native cell membranes, the SD-R150-T3L450B-10 cantilever series (Nanosensors, Neuchatel, Switzerland) was selected, with a tip radius of 150 nm, a resonance frequency of $\nu^{\prime }=$ 21 kHz, and a stiffness coefficient of K = 1 N/m.

The cantilever series SD-R150-T3L450B-10 (Nanosensors, Switzerland) was used to measure the Young’s modulus of native cell membranes, with a probe radius of 150 nm, resonance frequency, and a stiffness coefficient of K = 1 N/m. All AFM images and force curves were made using SPM Nova software (NT-MDT Spectrum Instruments, Russia).

### 2.9. Spectrophotometry

The experimental absorption spectrum of the RBC suspension, D(λ)_exper_, was recorded with a Unico 2800 spectrophotometer (United Products & Instruments, Dayton, OH, USA), taking measurements every 1 nm from 500 to 700 nm.

It was important for determining the true concentrations of the hemoglobin components directly in the bag. To do this, the optical absorption and scattering spectra of the cells were measured as the beam passed through a thin layer of pRBCs placed exactly inside the storage bag to prevent the conversion of deoxyhemoglobin to oxyhemoglobin in PBS solution that occurs immediately upon exposure to air. Any influence of plastic absorption and scatter in the 500–700 nm range was eliminated by calibration.

For suspension/lysate, 500 μL of solution was poured very rapidly (10 s) from a glass test tube into a quartz cuvette. The cuvette was closed with PARAFILM M (Pechiney Plastic Packaging, Chicago, IL, USA) before measurement in the spectrophotometer. Notably, the erythrocyte concentration in the glass tube was initially selected so that the suspension did not need to be diluted with PBS for spectrophotometer measurements.

We assessed the concentration of oxyhemoglobin (HbO_2_), deoxyhemoglobin (Hb), and methemoglobin (MetHb) in RBCs. To quantify these hemoglobin derivatives, we employed a nonlinear curve-fitting technique using Origin Pro 2019 (OriginLab Corporation, Northampton, MA, USA, software 9.8.0.200.). Our approach also takes into account scattering processes. More details can be found in our previous work [35].

To determine the hemolysis rate (%), we measured the spectra of the supernatant. The hemolysis percentage was derived using the following equation:

(1) $$\mathrm{Hemolysis}=\frac{C_{\sup}}{C_{\mathrm{sus}}}\cdot 100\%,$$

where $C_{\sup}$—total hemoglobin concentration in the supernatant, $C_{\mathrm{sus}}$—total hemoglobin concentration in the suspension.

### 2.10. Blood Test Methods

The acid–base status of the RBC suspension was analyzed using a STARTER 2100 (OHAUS, Parsippany, NJ, USA). An ST210 pH electrode (OHAUS, USA) was used. Biochemical analyzer AU 480 with accessories (Beckman Coulter, Inc., Brea, CA, USA) was used to determine the levels of lactate, glucose, and K^+^ throughout the storage period. The luciferin technique used the luminometer Zylux Corporation. An FB-12 (Bertold, Berlin, Germany) was used to measure the intracellular ATP concentration in erythrocytes.

### 2.11. Statistical Analysis

The statistical evaluation of the data was conducted with OriginPro 2019. The statistical figures for the samples are expressed as the mean along with the standard deviation (mean ± SD). To assess the significance of the disparities between the group averages, the Mann–Whitney nonparametric test was applied. A *p*-value of less than 0.05 was deemed to indicate significant differences. Furthermore, to estimate the quantity and average size of pores for a given sample size, we used Image Analysis P9 software (NT-MDT Spectrum Instruments, Russia).

## 3. Results

### 3.1. Effect of Xenon on the Change in Hemoglobin Components during pRBC Storage

In the first part of this study, the results of storage of bags containing pRBCs under standard conditions and with the inert gas xenon were compared. For this purpose, the bags of pRBCs were divided into two groups: Group A had no additive (referred to as control), and Group B was exposed to xenon (referred to as Xe) (Figure 1A).

**Figure 2:**
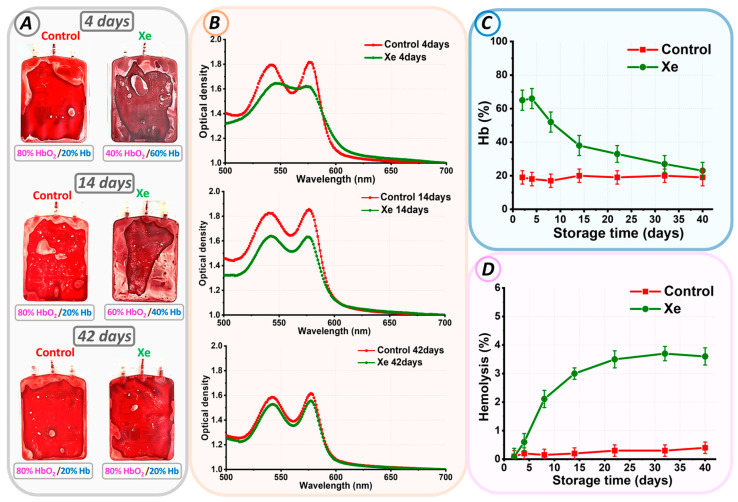
Changes in parameters of pRBCs during storage. (**A**) Photograph of control pRBCs and pRBCs exposed to Xe on days 4, 14, and 42. The ratio of oxygen to Xe as HbO_2_/Hb is indicated for each bag. (**B**) Optical spectra of erythrocyte suspensions of control pRBCs and pRBCs exposed to Xe at days 4, 14, and 42. (**C**) Alteration in Hb% concentration relative to storage duration (days). (**D**) Variation in hemolysis rate (%) over storage duration (days).

Initially, after the addition of xenon gas, the bags changed color from bright scarlet (control) to dark maroon (with Xe). As storage progressed, the bags with Xe became redder by day 14 (Figure 2A). The control bags remained bright scarlet.

Optical spectrophotometry was employed for the quantitative analysis of the observed effect. On each day of experimental storage, the optical absorption spectrum of hemoglobin was measured for the control and Xe groups. Figure 2B shows typical spectra for pRBCs stored under standard conditions (red line) and exposed to Xe (green line) for days 4, 14, and 42 of storage. The ratio of oxygen to Xe as HbO_2_/Hb is given below each image.

At the beginning of storage, the optical spectrum of control pRBCs matched that of oxyhemoglobin, showcasing two peaks at 542 nm and 577 nm. The addition of xenon to the pRBCs resulted in a change in the optical absorption spectrum of hemoglobin. In Figure 2B, the red spectrum (control) changed to a green spectrum (Xe).

Calculation of the percentage of hemoglobin components showed that in our experimental conditions, at the beginning of the storage period in bags with xenon, the amount of deoxyhemoglobin was 66 ± 6%, and in the control, it was 18 ± 4% (Figure 2). The formation of high concentrations of deoxyhemoglobin indicated exposure to xenon and the oxygen replacement it caused. Furthermore, the level of deoxyhemoglobin in the xenon bags decreased with time. For example, by day 14 of storage, it was 80%HbO_2_/20%Hb for the control and 60%HbO_2_/40%Hb for Xe. At the end of the storage period, the percentage of deoxyhemoglobin in the control and xenon bags was the same, i.e., 80%HbO_2_/20%Hb. However, no methemoglobin was formed in either sample.

The spectrophotometric method was used to calculate the level of free hemoglobin on each control day for all bags. The hemolysis rate (%) was calculated using Equation (1). In the control bags, the hemolysis rate did not exceed 0.8% by storage day 42. However, in the bags with added Xe, the situation was reversed. In such bags, the hemolysis rate was 3.6 ± 0.3% by the storage period’s end.

This difference in the hemolysis rate was related to the percentage ratio of oxygen and xenon. The functionality of both oxidant and antioxidant systems is influenced by the concentration of oxygen. As we have described previously [35], an optimal level of oxygen exists that promotes redox equilibrium. It is important to note that even a small shift in the ratio of gases, such as oxygen and xenon, from the optimal value will change the reactive oxygen species formation rate and, consequently, their levels. This, in turn, could significantly worsen the characteristics of the cells.

### 3.2. Changes in pRBC Morphology and Cytoskeleton during Storage

**Figure 3:**
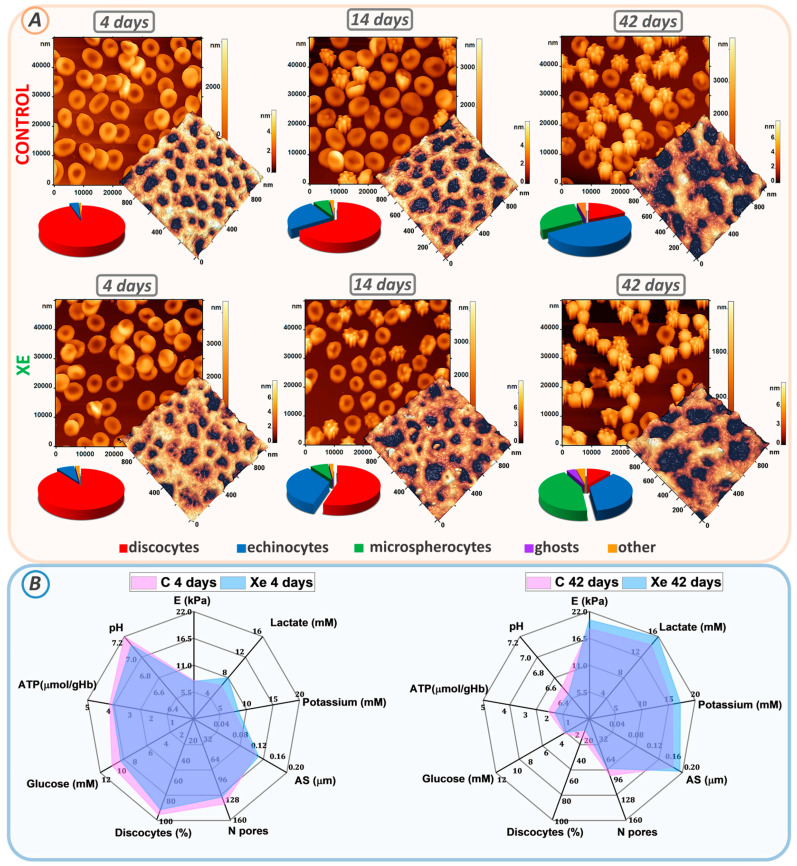
Changes in erythrocyte morphology, cytoskeletal structure, and biochemical parameters during storage. (**A**) AFM 2D images of control and Xe-exposed erythrocytes taken at days 4, 14, and 42. Each image is accompanied by AFM 3D images of 1 × 1 μm^2^ cytoskeletal fragments and a scatterplot of the cell count distribution of different cell types. (**B**) Radar plots comparing the percentages of biochemical parameters (lactate, potassium, glucose, ATP, and pH), biomechanical properties (E), cytoskeletal properties (AS, N-pores), and percentage of discocytes at days 4 and 42 in control and Xe-exposed samples.

Figure 3A shows the changes in erythrocyte morphology and cytoskeleton structure for control and pRBCs stored with xenon on days 4, 14, and 42 of storage.

At the beginning of storage, erythrocytes in both the control and Xe groups were found to be predominantly discocytes. The proportion of discocytes was 95 ± 6% in the control and 90 ± 7% in the Xe-exposed suspension. By the 14th day of storage, a transformation of some discocytes into echinocytes and microspherocytes was observed in both sets of samples. The control sample contained 25 ± 3% echinocytes and 7 ± 2% microspherocytes, while the Xe sample included 35 ± 4% echinocytes and 8 ± 3% microspherocytes.

Furthermore, a notable disparity in the count of echinocytes and discocytes was observed between the control and Xe-treated bags, with a significance level of *p* < 0.001. At the end of the storage period, the percentage of discocytes decreased to 19 ± 4% in the control bags and to 11 ± 4% in the Xe-exposed bags (*p* < 0.001) (Figure 3A). In the control bags, the remaining cells became echinocytes (48 ± 4%), microspherocytes (29 ± 4%), and other forms (4 ± 1%). In the bags with Xe, echinocytes (37 ± 4%), microspherocytes (44 ± 4%), other forms (4 ± 1%), and ghosts (4 ± 1%) also appeared.

The appearance of ghosts in the xenon bags was consistent with the observed hemolysis at the end of storage (Figure 2D). By the 42nd day of storage, most of the cells in the control bags had become microspherocytes and echinocytes. The number of microspherocytes and echinocytes in the control and xenon bags was significantly different at *p* < 0.001. This distribution of cell types is probably due to the amount of ROS generated during storage of the bags.

Cell morphology is determined by the structure of the cytoskeleton. Therefore, on each day of the experiment, the cytoskeleton was isolated, and its structure was examined. The cytoskeletal network consists of a pseudo-hexagonal meshwork containing filaments and pores between the filaments. Figure 3A demonstrates AFM 3D images of 1 × 1 μm^2^ sections of the cytoskeleton on days 4, 14, and 42 of storage, with filaments shown in the light and pores in the dark.

To quantify the change in pore size, we calculated the average pore size (AS) and the number of pores (N) in a 2.5 × 2.5 μm^2^ section using the Advance Watershed segmentation method of the Image Analysis P9 software. Figure 3B (Tables S1 and S2) shows the change in average pore size and number of pores from 4 to 42 days of storage. At the beginning of the storage period, the average AS pore size for the control bags was 0.134 ± 0.015 μm and 0.140 ± 0.016 μm for the Xe bags. Pore sizes were essentially unchanged by day 14 of suspension storage compared to day 4 of storage (*p* > 0.01). By day 42 of storage, the mean pore size was 0.180 ± 0.011 μm for the control and 0.195 ± 0.012 μm for the Xe. Moreover, the mean pore size for control and Xe differed significantly at *p* < 0.05.

Meanwhile, the number of N pores per unit area of the 2.5 × 2.5 μm^2^ scan decreased. On day 4 of storage, the average count of cytoskeletal pores within the 2.5 × 2.5 μm^2^ area reached 135 ± 22 in the control group and 124 ± 19 in the Xe group. By day 42 of storage, the mean number of pores decreased to 90 ± 14 in the control group and 79 ± 15 in the Xe group (Figure 3). In addition, the mean number of pores for the control and Xe groups did not differ significantly at the *p* > 0.05 level at the end of the storage period. In our earlier studies [27,36], we demonstrated that changes in cytoskeletal structure occur through the processes of protein filament disruption and clustering. In this experiment, samples with Xe had pores of similar size to the control, but their number was smaller. The protein filaments probably became more aggregated towards the end of the storage period in the presence of Xe, potentially accounting for the observed changes in cell shape. By day 42 of storage, the majority of cells in the control group had transformed into echinocytes, whereas the Xe group exhibited a greater percentage of both microspherocytes and echinocytes.

Cell deformability was evaluated using Young’s modulus E. At the start of the storage period, the mean Young’s modulus E for both the control and Xe groups was 7.9 ± 0.4 kPa and 7.7 ± 0.3 kPa, respectively. Cell stiffness increased to 18.5 ± 3.7 kPa for the control and 20.3 ± 3.4 kPa for Xe on day 42 of storage. The calculated mean Young’s modulus E for erythrocyte suspensions stored under standard conditions and those stored under xenon conditions did not differ at *p* > 0.05.

### 3.3. Parameters of RBC Preservative

Prolonged storage is associated with the development of oxidative stress, which manifests as a decrease in blood preservative pH, an increase in extracellular potassium concentration, a decrease in glucose level, a change in ATP level, an increase in lactate level, and a deterioration in blood biochemical parameters [37,38,39,40,41,42]. Figure 3B shows a comparison of these parameters at the beginning and end of storage in the control and Xe groups.

Prolonged storage resulted in a decrease in pH (from 7.2 ± 0.1 to 6.5 ± 0.1) in both the control and xenon bags (from 7.1 ± 0.1 to 6.4 ± 0.1) (Figure 3B, Tables S1 and S2). Cellular ATP decreased from 3.96 ± 0.40 µmol/g Hb to 1.93 ± 0.40 µmol/g Hb for the control and from 3.83 ± 0.50 µmol/g Hb to 1.63 ± 0.30 µmol/g Hb for Xe. Extracellular K^+^ increased to 15.7 ± 0.7 mM in the control and 17.2 ± 0.6 mM in the Xe group. By day 42 of storage, glucose concentration decreased from 10.5 ± 0.5 mM to 3.2 ± 0.3 mM for the control and from 9.3 ± 0.5 mM to 3.1 ± 0.3 mM for Xe, due to anaerobic metabolism. At the same time, lactate concentration increased to 14.4 ± 0.8 mM for the control and 15.8 ± 0.7 mM for Xe. These results correlate with similar findings obtained previously [27,28].

### 3.4. Changes in Absorption Spectra of Suspension and Lysate in Glass Tubes after Exposure to Xe

Because xenon diffuses through plastic in bags [43,44], it was decided to investigate its effects on RBCs using glass tubes. This allowed us to eliminate the influence of this factor on the experiment. The effect of xenon on erythrocytes is studied in the second part of our study by placing erythrocytes in a solution pre-saturated with xenon. PBS is used to make the suspension, and water is used for the lysate. This was important in order to understand which components of the cellular structure were affected by xenon. Such solutions will be referred to later in the text as PBS + Xe and H_2_O + Xe.

**Figure 4:**
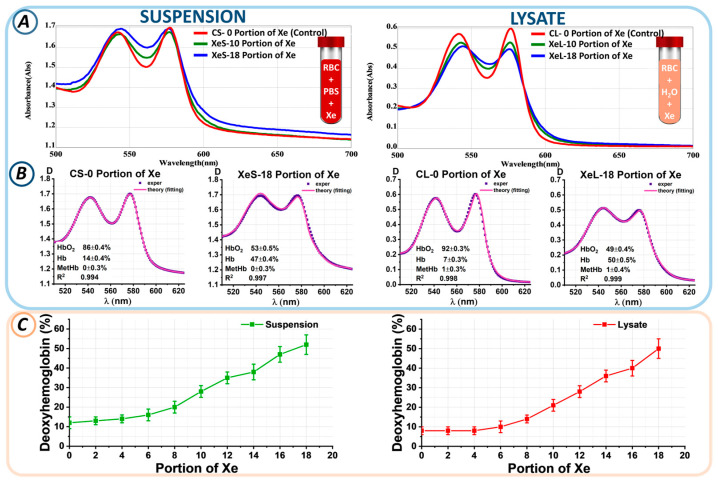
Changes in the levels of hemoglobin derivatives upon addition of a portion of Xe. (**A**) Optical spectra of suspension and lysate for the control sample and with different portions of Xe. (**B**) Example of fitting experimental data at 0 and 18 portions of Xe in RBC suspension and lysate. Here, 0 portions of Xe in suspension and lysate are labeled CS-0 portion of Xe and CL-0 portion of Xe (control samples), respectively; 18 portions of Xe in suspension and lysate are labeled XeS-18 portion of Xe and XeL-18 portion of Xe (xenon samples), respectively. The concentrations of hemoglobin derivatives shown in each graph were determined using the nonlinear curve-fitting method. (**C**) Change in Hb concentration (%) as a function of Xe portion in suspension and lysate.

The optical spectra of both the suspension and the lysate changed after exposure to xenon. The optical spectra in Figure 4A correspond to different portions of xenon in the suspension and lysate. The red curve represents the control. The amplitude of the peaks of the blue and green curves changed after the addition of PBS + Xe or H_2_O + Xe solution to RBCs (Figure 4A). In this case, HbO_2_ was partially converted to Hb. The effect was comparable for the suspension and the lysate.

Next, the proportions of hemoglobin derivatives within the suspension/lysate were measured through nonlinear curve fitting. Experimental spectral data, D(λ)_exper_ (blue), and the theoretical fitting curves, D(λ)_theory_ (pink), which most accurately represent the experimental findings, are displayed in Figure 4B. The calculated concentrations of three hemoglobin derivatives (Hb, HbO_2_, and MetHb) are shown in each plot. The approximation results are shown as value ± SE.

The control CS-0 suspension without xenon was characterized by a predominance of oxyhemoglobin at 83 ± 5%, while the deoxyhemoglobin percentage was 17 ± 4%. Exposure to 18 portions of xenon (XeS-18) resulted in an increase in deoxyhemoglobin to 52 ± 5%. Similar changes were observed in the lysate. In the CL-0 control sample, the percentage of HbO_2_ was 90 ± 5%, while that of Hb was 10 ± 3%. After exposure to 18 portions of xenon (XeL-18), the Hb level became 50 ± 5%. Similarly, the percentages of hemoglobin derivatives for each xenon portion were calculated.

Figure 4C shows the changes in the percentage of deoxyhemoglobin from the portion of Xe for suspension and lysate. We found that the higher the portion of Xe, the higher the level of deoxyhemoglobin. Thus, for the 10th portion of Xe, the deoxyhemoglobin level became 28 ± 3% in the suspension and 21 ± 3% in the lysate; for the 14th portion of Xe, 38 ± 4% in the suspension and 36 ± 3% in the lysate; and for the 18th portion of Xe, 52 ± 5% and 50 ± 5% in the suspension and lysate, respectively. Thus, the level of deoxyhemoglobin was a marker of xenon-induced oxygen displacement.

### 3.5. Changes in Erythrocyte Suspension Parameters during Storage

**Figure 5:**
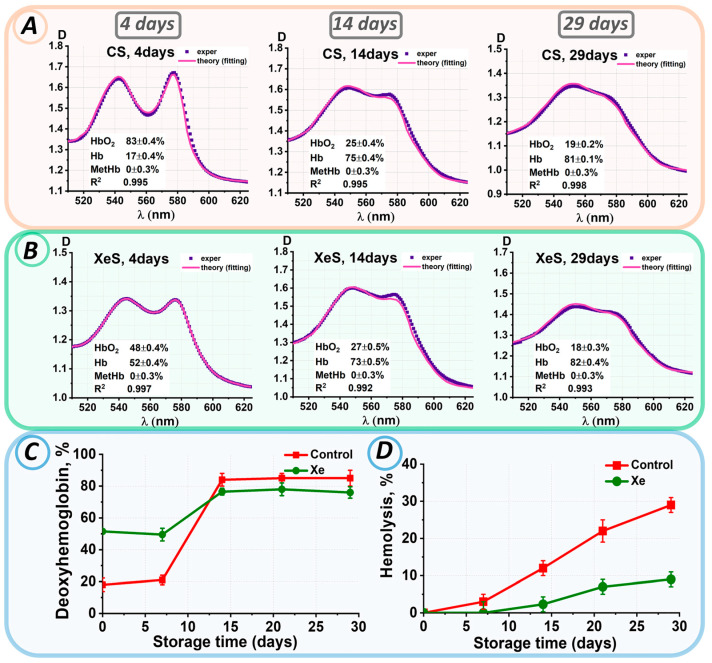
Changes in the characteristics of the erythrocyte suspension during storage. Analysis of experimental data results: (**A**) for control samples and (**B**) for samples with added xenon at 0, 14, 29 days. The concentrations of hemoglobin derivatives were estimated by the nonlinear curve-fitting approach and are presented in each graph as value ± SE. (**C**) Change in Hb concentration (%) during storage in control and xenon-exposed samples. (**D**) Change in % hemolysis for control and xenon-exposed samples.

During our experiment, suspensions without xenon and samples containing xenon were stored for 29 days. The levels of hemoglobin derivatives were assessed on each experimental day. For instance, Figure 5A,B display the experimental spectra of erythrocyte suspensions and the corresponding theoretical fitting curves on storage days 0, 14, and 29.

The deoxyhemoglobin concentration in both the control and xenon samples did not change during the first 8 days of storage, for the control up to 8 days Hb = 19 ± 3%, for the xenon samples up to 8 days Hb = 51 ± 4%. During the next seven days, there was a significant increase in the percentage of deoxyhemoglobin to 84 ± 4% for the control and 77 ± 2% for the xenon groups (Figure 5C). After 14 days of storage, the deoxyhemoglobin concentration remained at 81 ± 5% in all samples, which was completely different from the pattern of deoxyhemoglobin concentration changes in the pRBC bags (see Figure 2C).

Meanwhile, the percentage of MetHb remained unchanged with C(MetHb) = 0 ± 0.3% during the whole storage period.

Similar alterations in the concentrations of hemoglobin derivatives were observed in the lysate.

After 14 days of storage, a change in the color of the sample supernatants was observed, indicating hemolysis. Figure 5D shows the changes in hemolysis levels during storage in the control and xenon samples.

On day 14 of storage, hemolysis was present in 12 ± 2% of the control samples, whereas it was observed in approximately 2.3 ± 2% of the xenon samples. In both samples, hemolysis increased steadily over the next few storage days. Thus, by the 29th day of storage, hemolysis was 29 ± 2% in the control group. The hemolysis rate was three times slower in the xenon-exposed samples. On day 29 of storage, hemolysis in the xenon samples did not exceed 9 ± 2%. Thus, erythrocytes were less hemolyzed during storage of suspensions within glass tubes, which had tightly closed lids, in the presence of xenon compared to the control samples.

### 3.6. Effect of Oxygen on the Reversibility of Hemoglobin Derivatives

**Figure 6:**
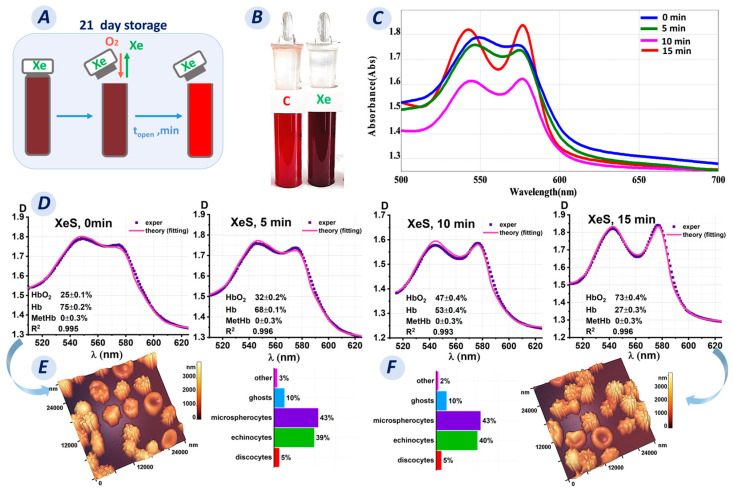
Transformation of hemoglobin derivatives upon opening a test tube after incubation for 21 days of storage. (**A**) Schematic representation of the change in oxygen concentration in a test tube after opening on day 21. (**B**) Photograph of the control tube (not saturated with Xe) and the tube with Xe on day 21. (**C**) Optical spectra as a function of the time of opening the tube containing Xe. (**D**) Fitting results of the experimental data for the Xe-exposed sample after opening at 5, 10, and 15 min. (**E**,**F**) AFM 3D images and graphs plots showing the number of cells with different shapes in the Xe-exposed samples before opening the tube and 15 min after opening.

When erythrocytes were added to a buffer/distilled water saturated with xenon, the optical spectra of the samples changed. This was primarily due to oxyhemoglobin conversion to deoxyhemoglobin, as evidenced by the color change of the suspension shown in Figure 6B and the calculated hemoglobin derivative concentrations in these samples.

Saturation of PBS/water with Xe led to deoxygenated hemoglobin formation in the RBCs. However, the question is whether this process is reversible and how long does the oxygenation process last?

Oxygenated hemoglobin was obtained by exposure to ambient oxygen. The tubes were opened and left for 0, 5, 10, and 15 min (Figure 6A). The spectra of the sample were then measured. The results obtained are shown in Figure 6C,D.

For our research, we collected samples on day 21 of storage. The Xe samples contained 75% deoxyhemoglobin, and the suspension was dark maroon in color (Figure 6B). We observed the reverse conversion of deoxyhemoglobin to oxyhemoglobin after opening the tubes. The optical spectrum of the Xe suspension changed from blue (for 0 min) to red (for 15 min), as shown in Figure 6C. In addition, the concentration of deoxyhemoglobin decreased every 5 min after opening the tubes: the C(Hb) percentage was 68% after 5 min, 53% after 10 min, and 27% after 15 min.

Deoxyhemoglobin concentrations changed similarly in the lysate. The percentage of deoxyhemoglobin was 61% prior to opening the tube. The C(Hb) percentage was 51% after 5 min, 27% after 10 min, and 8% after 15 min. As a result, oxyhemoglobin concentration increased. Methemoglobin formation was not observed.

Meanwhile, it was unclear what was happening to the morphology of erythrocytes at that time. Was the morphological change reversible like that of the hemoglobin derivatives, or were these processes unrelated?

The obtained AFM 3D images showed that on day 21 of storage, most of the cells were echinocytes (39 ± 2%) and microspherocytes (43 ± 2%), while discocytes (5 ± 1%), ghosts (10 ± 1%), and other shapes (3 ± 1%) were also observed (Figure 6E). Fifteen minutes after opening, the cell shape did not change significantly (*p* > 0.05) (Figure 6F), with the following percentages: discocytes, 5 ± 1%; echinocytes, 40 ± 2%; microspherocytes, 43 ± 2%; ghosts, 10 ± 1%; and other shapes, 2 ± 1%.

Remarkably, only 15 min of exposure to ambient oxygen was required to reduce the hemoglobin derivatives to oxyhemoglobin. However, this time was insufficient to restore the cell shape.

### 3.7. Changes in RBC Morphology

**Figure 7:**
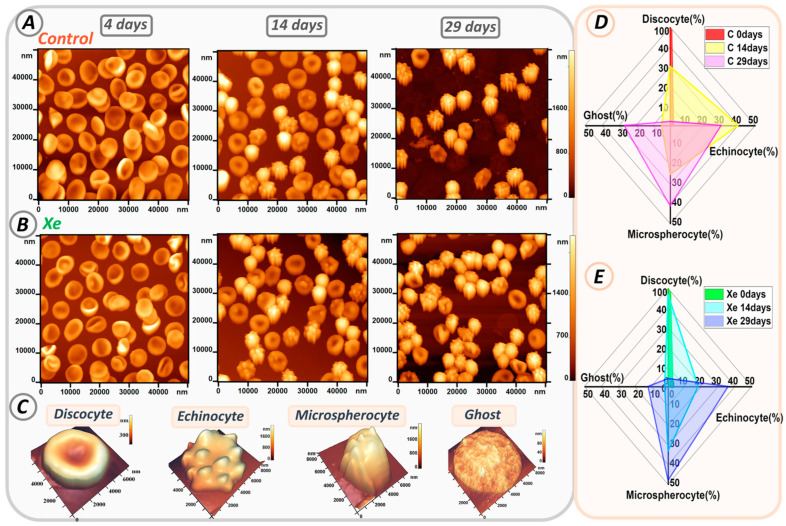
Alterations in erythrocyte shape during storage in glass tubes. (**A**) AFM 2D images of cells from control samples on days 0, 14, and 29. (**B**) AFM 2D images of cells from Xe-exposed samples on days 0, 14, and 29. (**C**) Typical cell shapes. (**D**,**E**) Radar plot showing the percentage of typical cell shapes in control and Xe-exposed samples on days 0, 14, and 29.

The morphology of erythrocytes in both control and Xe-exposed samples was studied throughout the time of storage in glass tubes. Figure 7A,B shows AFM 2D images of cells, 50 × 50 µm^2^ in size, from control samples and samples with added Xe. Storage of erythrocyte suspensions resulted in changes in cell shape. Typical cell shapes observed in the smears are shown in Figure 7C.

At the beginning of storage, discocytes were the most abundant in both samples, accounting for 98 ± 2% in the control and 97 ± 3% in the xenon group. However, their number decreased during storage as they transformed into different types of erythrocytes such as echinocytes, microspherocytes, and ghosts.

Figure 7D,E shows the evolution of each erythrocyte type over time for all samples. On the fourteenth day of storage in the control group, the number of discocytes decreased to 30 ± 5%, while echinocytes accounted for 40 ± 4%, microspherocytes for 25 ± 2%, and ghosts for 5 ± 1% (shown in yellow in Figure 7D). In contrast, samples with xenon contained 47 ± 5% discocytes, 18 ± 4% echinocytes, 33 ± 3% microspherocytes, and 2 ± 1% ghosts (shown in blue in Figure 7E).

By day 29 of storage, the majority of cells in both samples had transformed into echinocytes and microspherocytes. In the control group, there were 30 ± 4% echinocytes and 41 ± 3% microspherocytes, whereas in the Xe group, there were 36 ± 3% echinocytes and 48 ± 3% microspherocytes. The numbers of microspherocytes and echinocytes differed significantly between control and Xe tubes (*p* < 0.01).

In addition, the number of ghosts increased by 27 ± 4% in the control samples, consistent with the percentage of hemolysis shown in Figure 5D. Notably, samples with Xe had a lower number of ghosts (12 ± 3%) and a lower degree of hemolysis.

## 4. Discussion

In this study, we examined the comprehensive effects of the noble gas xenon on red blood cells. The study was divided into two main parts. The first part investigated the direct effect of xenon on erythrocytes by adding it to pRBC bags. The second part focused on comparing the response of erythrocytes to xenon in suspension and lysate in glass tubes with tightly closed lids.

Irreversible changes were observed in both the control and xenon-exposed samples during long-term storage of erythrocytes in bags. These changes were manifested as a change in the shape of the erythrocytes. By day 42, discocytes had irreversibly transformed into spheroechinocytes and spherocytes, ultimately becoming erythrocyte ghosts. This pattern of cell shape changes is indicative of erythrocyte aging and prolonged storage of donor erythrocytes [27,28,35,45,46].

As reported in our previous publications and other studies [27,45,46,47,48], the observed changes in cell shape may be associated with damage to local membrane and cytoskeletal connections. The cytoskeleton comprises a network of proteins, predominantly spectrin tetramers, linked with complexes of actin, protein 4.1R, ankyrin, as well as tropomyosin, tropomodulin, adducin, and dematin [48,49]. The integrity and flexibility of the membrane is largely maintained by the interaction of the spectrin-ankyrin band 3 complex [47,50]. In addition, disruption of these bonds can cause spectrin tetramers to dissociate into dimers [51], and the protein structures of the matrix network can undergo polymerization and aggregation [52]. In the present study, we observed rearrangement of the cytoskeletal network during the storage of pRBCs: cytoskeletal filaments were deformed and ruptured, small pores merged into larger entities, and clustering of protein complexes occurred (Figure 3). The transformation of discocytes into other cell types is caused by changes in the cytoskeleton [27,28].

In addition, a 2.5-fold increase in membrane stiffness was observed. The aggregation of band 3 protein results in decreased affinity for the cytoskeletal complex, which in turn increases membrane stiffness and reduces cellular functionality [47,50,53]. Erythrocyte stiffness is an important blood flow parameter because stiffer erythrocytes have more difficulty passing through small capillaries to deliver oxygen.

Based on the observed changes, we believe that the main cause of cell membrane damage and erythrocyte dysfunction is the activation of oxidative processes. In our study, pH dropped to 6.4 by day 42 of storage, cellular ATP and glucose concentrations decreased, and lactate and extracellular potassium levels increased. These data are consistent with the results of other studies [37,38,39,40,41,42,45,54,55,56].

The high concentration of molecular oxygen in hemoglobin and the presence of polyunsaturated fatty acids in the cell membrane increase the risk of lipid peroxidation. Oxidative processes in erythrocytes are characterized by the fact that oxygen released from the hemoglobin molecule carries an electron with it, generating reactive oxygen species [57,58]. These processes are regulated by interactions between the oxidative and antioxidant systems of cells. An imbalance between these systems can lead to oxidative stress, which, in turn, can cause structural and functional abnormalities in erythrocytes.

The main difference between the control and Xe samples was the increase in the level of deoxyhemoglobin. When the inert gas Xe was added to the pRBC samples, the conversion of oxyhemoglobin to deoxyhemoglobin occurred and reached 60%. However, when stored in bags with added xenon, the deoxyhemoglobin level decreased to the control level.

What causes these changes in deoxyhemoglobin? This may be due to the special plastic bags in which the pRBCs are stored. Xenon is known to diffuse through plastic [43,44]. It is likely that Xe diffused through the bag in our study, causing the level of deoxyhemoglobin to decrease with each day of storage. Therefore, it was decided to perform the second part of the study in glass tubes with sealed lids to eliminate the influence of this factor on the experiment.

In the second part of the study, erythrocytes showed a pattern of morphological changes similar to that observed during bag storage. In particular, the formation of deoxyhemoglobin was the main difference between the samples exposed to Xe. The concentration of deoxyhemoglobin correlated with the amount of Xe added (Figure 5C). In fact, the higher the portion of Xe, the more deoxyhemoglobin was detected. This effect was observed both when cells were exposed to xenon and when it was added to hemolysate, i.e., directly to hemoglobin.

Despite the chemical inertness of xenon, there is evidence of its biological activity [59]. On the one hand, the unique properties of xenon allow it to diffuse and interact with cellular structures at the molecular level. On the other hand, xenon is believed to be able to interact with proteins that have complex structures and contain various cavities and microsites. These microsites are known as “xenon-binding pockets” [59,60,61,62]. Interestingly, this interaction is not static, and these pockets are not occupied by xenon alone. Research [63] has shown that xenon can migrate through channels in myoglobin, resulting in a dynamic interaction.

It was also found that increasing the oxygen level in the test tube caused the conversion of deoxyhemoglobin back to oxyhemoglobin. However, the shape of the cells did not return to their original discocyte form. It is likely that the changes in cell shape were a result of the storage conditions rather than the effect of xenon on the erythrocyte membrane. Research conducted by [64] suggested that deoxyhemoglobin might affect the interaction between band 3 and the RBC cytoskeleton. It was demonstrated that deoxygenating red blood cells disrupts the ankyrin bridges of band 3, resulting in the detachment of the spectrin/actin cytoskeleton from the membrane. Prolonged periods of oxygen deprivation can cause the release of small membrane vesicles. Therefore, it is possible that the persistent existence of erythrocytes in the deoxyhemoglobin form may contribute to alterations in erythrocyte shape.

It was also found that both experimental groups had a 5% hemolysis rate when exposed to Xe. In contrast, the control group consisting of pRBCs had a hemolysis rate of only 0.5%. Interestingly, the opposite results were observed when erythrocytes were stored in sealed tubes without oxygen. The control samples experienced a significantly higher rate of hemolysis (up to 30%). Therefore, it is critical to carefully adjust the concentration of oxygen and xenon in the mixture to minimize erythrocyte damage. Determining the optimal oxygen level can help balance the oxidative and antioxidant systems and reduce the risk of hemolysis.

We have evaluated the potential protective effects of xenon on erythrocytes during long-term storage. Although xenon, as an inert gas, does not cause oxidative stress, changes in the gas composition caused by its addition may create situations that enhance oxidative processes.

These results highlight the importance of further research into the use of inert gases to slow cell aging under storage conditions. The permeability of inert gases through plastic bags is a critical factor to consider. In addition, the determination of the optimal xenon/oxygen mixture or its use on key days of storage is essential. The effects of donor sex/age and blood group on these processes should also be investigated.

## 5. Conclusions

Our study showed that when erythrocytes were exposed to xenon during storage, they underwent significant structural changes similar to those observed under normal storage conditions. These changes include irreversible cell transformations such as the formation of echinocytes, microspherocytes, and ghosts. In bags of pRBCs, the level of hemolysis during storage with xenon was six times higher than the acceptable limit. However, in the sealed glass tube experiment, hemolysis of samples exposed to xenon was minimal. A specific effect of xenon exposure was the production of deoxyhemoglobin in both the cell suspension and the hemolysate. Unfortunately, our study did not show any apparent protective effects of xenon. It is possible that xenon concentration is a critical factor. Therefore, it is important to conduct further research to determine the optimal concentration of xenon to improve the properties of stored erythrocytes.

## Data Availability

The datasets used and analyzed during the current study are available from the corresponding authors upon request.

## Supplementary Materials

The following supporting information can be downloaded at: https://www.mdpi.com/article/10.3390/cells13050411/s1. Table S1 shows a comparison of the percentages of biochemical parameters (lactate, potassium, glucose, ATP, and pH), biomechanical properties (E), cytoskeletal properties (AS and N-pores), and percentage of discocytes at day 4 in control and Xe-exposed samples. Table S2 shows a comparison of the percentages of biochemical parameters (lactate, potassium, glucose, ATP, and pH), biomechanical properties (E), cytoskeletal properties (AS and N pores), and percentage of discocytes at day 42 in control and Xe-exposed samples.
