# Supplementary material for: Red Blood Cell Storage with Xenon: Safe or Disruption?
Source: Cells. 2024 Feb 27;13(5):411. doi: 10.3390/cells13050411 (PMC10930635; doi:10.3390/cells13050411)
Supplement: Supplementary file 1 [file cells-13-00411-s001.zip › cells-2845589-supplementary.pdf]

Table S1: Table comparing the percentages of biochemical parameters (lactate, potassium, glucose, ATP, pH), biomechanical properties (E), cytoskeletal properties (AS, N pores), and percentage of discocytes at day 4 in control and Xe-exposed samples.

Parameter	Control	Xe
E (kPa)	7.9±0.4	7.7±0.3
Lactate (mM)	6.48±0.6	8±0.7 ***
Potassium (mM)	7.6±0.4	8.45±0.6 **
AS (μm)	0.134 ± 0.015	0.140 ± 0.016
N pores	135 ± 22	124 ± 19
Discocytes (%)	95 ± 6	90 ± 7
Glucose (mM)	10.5 ± 0.5	9.3 ± 0.5 ***
ATP (μmol/gHb)	3.96 ± 0.4	3.83± 0.5
pH	7.2 ± 0.1	7.1 ± 0.1

Statistical significance of the obtained values was tested the Mann-Whitney nonparametric test was applied (** $p < 0.01$, *** $p < 0.001$ compared with control sample at day 4).

Table S2: Table comparing the percentages of biochemical parameters (lactate, potassium, glucose, ATP, pH), biomechanical properties (E), cytoskeletal properties (AS, N pores), and percentage of discocytes at day 42 in control and Xe-exposed samples.

Parameter	Control	Xe
E (kPa)	18.5±3.7	20.3±3.4
Lactate (mM)	14.4±0.8	15.8±0.7 **
Potassium (mM)	15.7± 0.7	17.2±0.6 ***
AS (μm)	0.180 ± 0.011	0.195 ± 0.012 *
N pores	90 ± 14	79 ± 15
Discocytes (%)	19 ± 4	11 ± 4 ***
Glucose (mM)	3.2 ± 0.3	3.1± 0.3
ATP (μmol/gHb)	1.93 ± 0.4	1.63 ± 0.3
pH	6.5 ± 0.1	6.4 ± 0.1

Statistical significance of the obtained values was tested the Mann-Whitney nonparametric test was applied (* $p < 0.05$; ** $p < 0.01$, *** $p < 0.001$ compared with control sample at day 42).
